# Myosteatosis as a New Risk Factor of Surgical Complications in Kidney Transplant Recipients: A Retrospective Study

**DOI:** 10.1002/jcsm.13746

**Published:** 2025-04-30

**Authors:** Antoine Morel, Yaniss Ouamri, Lauriane Ségaux, Louai Zaidan, Michael Moryoussef, Sébastien Mulé, Cécile Maud Champy, Edouard Reizine, Alexandre Ingels, Alain Luciani, Philippe Grimbert, Florence Canouï‐Poitrine, Marie Matignon, Frédéric Pigneur, Thomas Stehlé

**Affiliations:** ^1^ Université Paris Est Créteil, Institut National de la Santé et de la Recherche Médicale (INSERM), Institut Mondor de Recherche Biomédicale (IMRB) Créteil France; ^2^ Assistance Publique des Hôpitaux de Paris (AP‐HP) Hôpitaux Universitaires Henri Mondor, Service de Néphrologie et Transplantation, Fédération Hospitalo‐Universitaire “Innovative therapy for immune disorders” Créteil France; ^3^ Assistance Publique des Hôpitaux de Paris (AP‐HP) Hôpitaux Universitaires Henri‐Mondor, Service d’Imagerie Médicale Créteil France; ^4^ Assistance Publique des Hôpitaux de Paris (AP‐HP) Groupe Hospitalier Henri‐Mondor/Albert Chenevier, Clinical Epidemiology and Ageing Unit (CEpiA) Créteil France; ^5^ Assistance Publique‐Hôpitaux de Paris (AP‐HP) Hôpitaux Universitaires Henri Mondor, Service d’Urologie, Groupe Hospitalier Henri‐Mondor/Albert Chenevier Créteil France

## Abstract

**Background:**

Computed tomography (CT) scan–defined myosteatosis is a common feature in ESKD patients receiving kidney transplantation (KT) and is associated with mortality after KT. We aimed to explore the impact of myosteatosis and other CT scan based morphometric data on the occurrence of early surgical complications after KT.

**Methods:**

We retrospectively measured on an unenhanced cross‐sectional CT scan taken at the middle of the third lumbar vertebra performed within the previous year or at the time of KT: surface muscle index (total lumbar cross‐sectional muscle area [CSMA] divided by height squared), subcutaneaous adipose tissue index, visceral adipose tissue index and muscle density (MD: mean CT attenuation of CSMA). Vessel to skin distance was the distance between iliac vein and skin. Myosteatosis was defined as MD below age‐ and sex‐specific normal values. Logistic regression models were constructed to identify predictive factor of 90 days postoperative surgical complications with Clavien–Dindo score greater than or equal to 2, CD ≥ 2).

**Results:**

Among the *N* = 200 patients, 61.5% were male with a mean age of 54.8 (± 13.8) years and a mean BMI of 25.1 (± 4.4) kg/m^2^. Sixty patients (30%) developed at least one postoperative complication (CD ≥ 2) in the first 3 months after KT. In two different multivariate analyses, MD (aOR: 0.95 for one Hounsfield unit increase, 95% CI: 0.91–0.99, *p* = 0.028) and myosteatosis status (aOR: 4.64, 95% CI: 2.18–9.90, *p* < 0.0001) were the only independent risk factors for postsurgical complication.

**Conclusions:**

Myosteatosis is independently associated with the occurrence of CD ≥ 2 postoperative complication within 90 days of surgery.

## 1 Introduction

Kidney transplantation (KT) is the kidney replacement therapy (KRT) method with the best survival in patients with end‐stage kidney disease (ESKD), compared with peritoneal dialysis or haemodialysis, even in older patients (e.g., > 65 years) engrafted from expanded criteria donors (ECD) [1, 2]. However, the first years after KT, especially for older kidney transplant recipients (KTr), represent a period when the risk for mortality is higher than during dialysis therapy [1, 2]. Initial excess risk is related to conditioning for surgery (e.g., anaesthesia), immunosuppressive induction treatment but especially the surgical event itself [3]. Surgical morbidity of KT is related to well‐known different variables such as age, body mass index and surgical techniques (e.g., open surgery vs robot‐assisted) [3, 4, 5]. Postoperative morbi‐mortality remains significant in KTr despite an improving trend during the last decade [6]. Therefore, the search for new risk factors for early surgical complications seems crucial to improve care for these patients.

Muscle mass, muscle quality and other morphometric data such as subcutaneous and visceral adipose tissues and vessel‐to‐skin distance (VSK) are now easily assessable from computed tomography (CT) scan imaging [7, 8]. Analysis of non‐enhanced abdominal CT sections at the third lumbar vertebra (L3) provides surrogate markers for skeletal muscle mass and myosteatosis, that is fat infiltration within skeletal muscles. Total skeletal muscle mass can be estimated with skeletal muscle index (SMI), defined as total lumbar cross‐sectional muscle area (CSMA) through the middle of L3 divided by height squared, and mean skeletal muscle density (MD) of CSMA can be used for estimating myosteatosis [7]. In the field of surgery, myosteatosis is associated with higher morbidity, especially in liver transplantation [9], oesophageal, gastric cancer [10] and in colorectal cancer surgery [11]. CT scan defined myosteatosis might be frequent in ESKD patients receiving KT, with up to 25% of KTr having MD below the 2.5th percentile of age and sex reference values. In addition, myosteatosis in KTr would be associated with long‐term mortality [12, 13, 14]. However, only limited data are available on the relationship between MD and short‐term outcomes of KT, in particular morbidity surrounding surgery. We therefore aimed to investigate the association between CT‐based morphometric data and early surgical complications after KT.

## 2 Methods

### 2.1 Study Design

We conducted a single‐centre retrospective study to explore the impact of different CT scan‐based morphometric data on the occurrence of early surgical complications after KT (within 90 days after KT).

### 2.2 Setting and Population

This retrospective study was conducted on a previously published cohort (*N* = 200 patients) [12]. All consecutive patients who underwent KT in our institution, between 1st of January 2014 and 31st of December 2017, who had an abdominal CT scan performed within 1 year prior to KT and up to 14 days after, and for whom CT scan images were available in our hospital medical picture archiving and communication system (PACS), were considered for inclusion. Apart from the unavailability of these CT images, there were no exclusion criteria. The Institutional Review Board ‘Comité de Protection des Personnes, Ile de France IV’ approved this single‐centre study (IRB #00003835). Informed consents were obtained from all the participants beforehand. The research was conducted in accordance with the Declaration of Helsinki, and clinical and research activities being reported are consistent with the Principles of the Declaration of Istanbul as outlined in the ‘Declaration of Istanbul on Organ Trafficking and Transplant Tourism’.

### 2.3 Variables

Recipients' characteristics, donor demography, immunological KT characteristics (including donor‐specific anti‐HLA antibodies and immunosuppressive therapies), delay graft function, intra and postoperative outcomes, allograft loss and death were all recorded in our local database.

Surgical complications were defined as any surgical complications observed during the first 90 days following KT according to the Clavien–Dindo classification (CD) [15]. Clavien–Dindo classification was defined as:

- Grade I: Any deviation from normal postoperative course without the need for pharmacological treatment or surgical, endoscopic and radiological interventions.
- Grade II: Requiring pharmacological treatment with drugs other than such allowed for Grade I complications (blood transfusions and total parenteral nutrition are also included).
- Grade III: Requiring surgical, endoscopic or radiological intervention (IIIa: not under general anaesthesia; IIIb: under general anaesthesia).
- Grade IV: life‐threatening complication requiring intensive care unit management (IVa: single‐organ dysfunction including dialysis, IVb: multiorgan dysfunction).
- Grade V: death of the patient.
- Early urinary tract infections within 90 days of KT were excluded. Indeed, there is a wide diversity of risk factors for early urinary tract infection, including the presence of a double‐J stent during the first month after KT, making the correlation between surgery and this infectious complication uncertain [16].

Delayed graft function (DGF) was defined as the need of dialysis within 7 days after transplantation [17]. Allograft loss was defined as the need for long‐term dialysis and/or retransplantation [17].

Intraoperative complications were defined as complications occurring during surgery, while postoperative complications were defined as complications occurring after surgery and up to 90 days following KT.

### 2.4 Morphometric Measures

**FIGURE 1:**
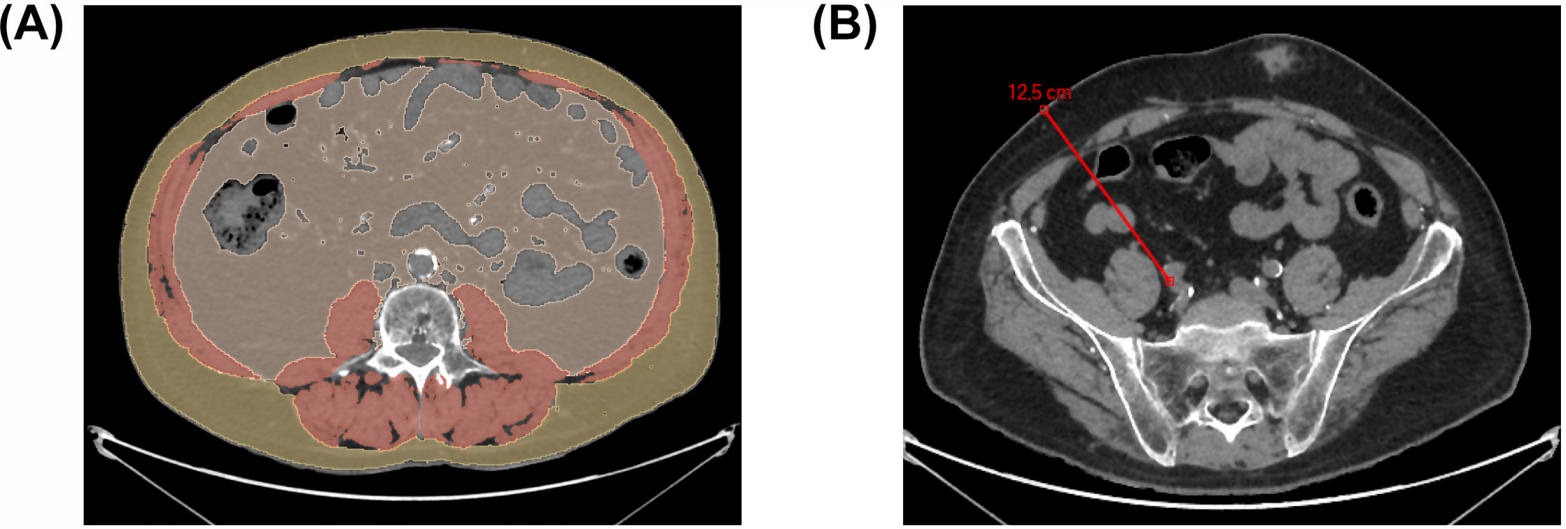
(A) Cross‐sectional computed tomography images in a kidney transplant recipient after muscle and fat segmentation at the level of the third lumbar vertebra. Skeletal muscle area (red) was identified by using computed tomography attenuation values of −29 to +150 Hounsfield units (HU). Subcutaneous fat area (yellow) was depicted by using attenuation values of −190 to −30 HU. For visceral fat area (orange), −150 to −50 HU attenuation values were used. (B) Vessel‐to‐skin (VSK) distance measurement on abdominal CT scan. VSK was defined by the distance between the anterior edge of the iliac vein and the skin, passing through the semilunar line.

All CT imaging were performed using one of the three multi‐detector CT scans in our radiology department: Discovery CT®, Revolution CT® and Lightspeed VCT® (GE Healthcare, Milwaukee, WI, USA), according to a standardised protocol as previously depicted [12], with the following CT imaging parameters: unenhanced acquisition, 120 kV voltage, filtered back projection image reconstruction without iterative reconstruction, 1.25 mm contiguous reconstructed slice thickness and soft filter kernel [12]. Segmentations were performed using a dedicated posttreatment station (Advantage Window v4.7; GE Healthcare, Buc, France). Pre‐established attenuation threshold (−29 to +150) Hounsfield unit (HU) was selected to segment semi‐automatically the CSMA [18], including the external and internal obliques, paraspinal, rectus abdominis, transversus abdominis and psoas muscles areas on axial section passing through the middle of L3 [19]. Visceral fat area (VFA) and subcutaneous fat area (SFA) were segmented and quantified in square centimetre on the same section as the CSMA. Visceral adipose tissue was selected by using the attenuation threshold within −150 and −50 HU [18], whereas subcutaneous fat was selected between −190 and −30 HU [18, 20] (Figure 1A). CT segmentation was performed by Y.O., senior radiologist, expert in abdominal imaging with 8 years of experience.

#### 2.4.1 Surface Muscle Index (SMI), MD and Myosteatosis

CSMA was normalised for height squared and reported as the SMI in square centimetre per square meter [18]. MD was defined as the mean CT attenuation of CSMA at the middle of L3 and was expressed in HU [7]. To define myosteatosis (i.e., low‐MD), we used previously published age‐ and sex‐specific cutoffs from a local cohort of 130 healthy subjects [12].

#### 2.4.2 Subcutaneaous Adipose Tissue Index (SATI) and Visceral Adipose Tissue Index (VATI)

Subcutaneaous and visceral adipose areas at the middle of L3 were both normalised for height squared and reported as SATI and VATI, in square centimetre per square meter.

#### 2.4.3 Vessel to Skin (VSK) Distance

VSK distance was defined by the distance from the anterior edge of the iliac vein at the level of its bifurcation to the skin passing through the musculo‐aponevrotic line of the abdominal transverse muscle (Figure 1B) as usually referenced in the literature [8].

### 2.5 Study Endpoints

Primary clinical endpoint was the occurrence of CD ≥ 2 surgical complications during the first 90 days following KT. The reason for using the CD ≥ 2 threshold as the primary clinical criterion is that surgical complications requiring pharmacological treatment predominate and account for the bulk of postsurgical morbidity in kidney transplantation.

Secondary endpoints included (i) description of surgical complications according to the CD classification; (ii) determination, based on univariate then multivariate analysis, of risk factors for having a CD ≥ 2 complication after KT; the prevalence in the whole cohort and according to myosteatosis status of (iii) delayed graft function; (iv) surgical complications; (v) early allograft loss; and (vi) early death, 90 days after KT.

### 2.6 Statistical Analysis

Categorical data were expressed as numbers and percentages, and continuous variables were expressed as medians with interquartile ranges (IQR) or as means with standard deviations (SD), as appropriate. Continuous variables were compared between two groups using *t* test (or Mann–Whitney test) depending on the skewness of their distribution. Categorical variables were compared using chi‐square test (or Fisher exact test) as appropriate. Risk factors associated with the occurrence of a CD ≥ 2 complication within first 90 days after KT were evaluated using logistic regression analysis. Factors identified with a *p*‐value of < 0.2 in the univariate analysis were considered for the multivariate model, which was then subjected to stepwise backward elimination, with an exclusion threshold of 5%, until the final model was obtained. Results were expressed as crude odd ratios (cORs) and adjusted odd ratios (aORs) with 95% confidence intervals (95% CI). A first multivariate model has been made up using MD as a continuous variable. A second model has been built using MD as a categorical variable (i.e., myosteatosis) [12]. We excluded highly correlated variables in order to avoid multicollinearity effects. The accuracies of multivariate models including either myosteatosis status (qualitative) or MD as a quantitative variable were estimated using area under the ROC curve values; then, they were compared with a multivariate model including no CT data, using a likelihood ratio test. Two‐tailed *p* values of < 0.05 were considered statistically significant. All analyses and graphics were conducted on Stata v15.0 (StataCorp, College Station, TX, USA).

## 3 Results

### 3.1 Patient Characteristics

Among the *N* = 200 patients included between January 2014 and December 2017, 61.5% were male with a mean age of 54.8 (± 13.8) years and a mean BMI of 25.1 (± 4.4) kg/m^2^ **(**Table 1 **)**. KT was the first in 82% of them. Twenty‐one percent of patients experienced a major cardiovascular event before KT (i.e., coronary heart disease and/or peripheral artery disease and/or history of cerebral stroke), and 26.5% had diabetes mellitus. Recipients were engrafted from deceased donor in 87%, with extended criteria donors (ECD) in 55.5%. Almost all patients received induction immunosuppressive therapy (99.5%), mainly antithymocyte globulin (69%). Maintenance immunosuppressive regimen consisted mostly of a combination of calcineurin inhibitors (91%), mycophenolic acid (82.5%) and corticosteroids (100%) (Table 1). In the entire cohort, mean SMI were 49.7 (± 8.6) and 42.3 (± 7.3) cm^2^/m^2^, mean MD were 30.6 (± 9) and 29.7 (± 8.4) HU, mean VATI were 28.6 (± 60.6) and 18.6 (± 13.8) cm^2^/m^2^ and mean SATI were 19.4 (± 10.1) and 32.5 (± 16.5) cm^2^/m^2^ for male and female, respectively. Median VSK were 13.50 (11.1;15.7) and 12.6 (11.5;14.3) cm for male and female, respectively **(**Table 1 **)**.

**TABLE 1:** Recipient and donor baseline characteristics in the entire cohort and according to the occurrence of CD ≥ 2 surgical complication (CD ≥ 2) within 90 days after KT.

Variables	Whole cohort, *N* = 200	No/minor complications (CD < 2), *N* = 140	CD ≥ 2 complications, *N* = 60	*p*
Recipients characteristics				
Male, *N* (%)	123 (61.5%)	85 (60.7%)	38 (63.3%)	0.73
Age, years, mean ± SD	54.8 (± 13.8)	53.71 (± 14.1)	57.32 (± 12.8)	0.09
BMI (kg/m^2^), mean ± SD	25.1 (± 4.4)	25.19 (± 4.4)	25.0 (± 4.6)	0.75
BMI (kg/m^2^) > 25, *N* (%)	91 (45.5%)	66 (47.1%)	25 (41.7%)	0.48
Requiring KRT before KT, *N* (%)	181 (90.5%)	126 (90.0%)	55 (91.7%)	0.71
Duration of KRT, months, median (IQR) *N* = 181	49.0 (25.0; 71.0)	47.0 (25.0; 72.0)	51.0 (20.0; 68.0)	0.77
Diabetes mellitus, *N* (%)	53 (26.5%)	32 (22.9%)	21 (35.0%)	0.07
History of cancer, *N* (%)	28 (14.0%)	23 (16.4%)	5 (8.3%)	0.13
History of MCVE, *N* (%)	42 (21.0%)	26 (18.6%)	16 (26.7%)	0.20
Initial kidney disease				
Diabetes mellitus, *N* (%)	31 (15.5%)	21 (15.0%)	10 (16.7%)	0.77
Nephroangiosclerosis, *N* (%)	18 (9.0%)	11 (7.9%)	7 (11.7%)	0.39
Polycystic kidney disease, *N* (%)	20 (10.0%)	35 (25.0%)	14 (23.3%)	0.80
IgA nephropathy, *N* (%)	15 (7.5%)	15 (10.7%)	5 (8.3%)	0.61
Tubulo‐interstitial chronic nephropathy, *N* (%)	6 (3.0%)	9 (6.4%)	6 (10.0%)	0.38
Unknown, *N* (%)	49 (24.5%)	5 (3.6%)	1 (1.7%)	0.47
Other, *N* (%)	61 (30.5%)	44 (31.4%)	17 (28.3%)	0.66
Double KT, *N* (%)	9 (4.5%)	6 (4.3%)	3 (5.0%)	0.82
KT > 1, *N* (%)	36 (18.0%)	22 (15.7%)	14 (23.3%)	0.20
Ly counts < 1G/L within 7 days before KT, *N* (%)	66 (33.0%)	44 (31.4%)	22 (36.7%)	0.47
Albuminemia within 7 days before KT, median (IQR) *N* = 177	40.80 (38.0; 44.0)	41.0 (38.0; 43.3)	40.3 (37.8; 44.6)	0.90
CT scan data				
SMI (cm^2^/m^2^) male/female, mean ± SD	49.7 (± 8.6)/42.3 (± 7.3)	46.7 (± 8.3)	47.2 (± 10.2)	0.73
Muscle density (HU) male/female, mean ± SD	30.6 (± 9)/29.7 (± 8.4)	31.6 (± 8.3)	27.3 (± 9.1)	**0.001**
Myosteatosis male/female, *N* (%)	34/49 (69%)/15/49 (31%)	21 (15%)	28 (46.7%)	**< 0.0001**
VATI (cm^2^/m^2^) male/female, mean ± SD	28.6 (± 60.6)/18.6 (± 13.8)	25.5 (± 57.1)	22.9 (± 15.2)	0.72
SATI (cm^2^/m^2^) male/female, mean ± SD	19.4 (± 10.1)/32.5 (± 16.5)	24 (± 13.8)	25.4 (± 15.7)	0.54
Vessel to skin distance (cm) male/female, median (IQR)	13.5 (11.1; 15.7)/12.6 (11.5; 14.3)	11.6 (10.2; 14)	13.2 (10.5; 15.4)	**0.034**
Donor demography				
Deceased donor, *N* (%)	174 (87%)	121 (86.4%)	53 (88%)	0.71
Donor age, mean ± SD	57.6 (± 17)	56.6 (± 17.3)	59.8 (± 16.3)	0.21
Donor male, *N* (%)	108 (54%)	75 (53.6%)	33 (55%)	0.85
SCr donor, median ± SD	73.5 (59; 100.5)	74 (57.5; 99.5)	73.5 (61; 104)	0.82
ECD criterias, *N* (%)	111 (55.5%)	73 (52.1%)	38 (63.3%)	0.14
Cold ischemia, minutes, median (IQR)	1032.5 (819.5; 1277)	1042 (799.5; 1263.5)	1005 (905; 1280)	0.36
Immunological Kidney transplantation characteristics				
Donor‐specific anti‐HLA antibodies, *N* (%) (*N* = 190)	59 (29.5%)	47 (33.6%)	12 (20%)	0.09
Induction immunosuppressive therapy, *N* (%)	199 (99.5%)	139 (99.3%)	60 (100%)	1.00
Antithymocyte globulin, *N* (%)	138 (69%)	96 (68.6%)	42 (70%)	0.84
Maintenance immunosupressive therapy				
Calcineurin inhibitors, *N* (%)	182 (91%)	125 (89.3%)	57 (95%)	0.20
Belatacept, *N* (%)	19 (9.5%)	15 (10.7%)	4 (6.7%)	0.37
Mycophenolic acid, *N* (%)	165 (82.5%)	117 (83.6%)	48 (80%)	0.54
mTOR inhibitors, *N* (%)	32 (16%)	22 (15.7%)	10 (16.7%)	0.87
Steroids, *N* (%)	200 (100%)	140 (100%)	60 (100%)	1.00

*Note: p*‐value was calculated between no/minor surgical complications group and CD ≥ 2 surgical complications group, using a *χ* ^2^ test for categorial variables and *t*‐test for quantitative variables. *p*‐values < 0.05 are in bold.

Abbreviations: BMI, body mass index; ECD, extended‐criteria donor; HU, Hounsfield units (density); IQR, interquartile range; KRT, kidney replacement therapy; KT, kidney transplantation; Ly, lymphocyte; MCVE, major cardiovascular event (i.e., coronary heart disease and/or peripheral artery disease and/or history of cerebral stroke); SATI, subcutaneous adipose tissue index; sCr, serum creatinine concentration; SMI, skeletal muscle mass index; VATI, Visceral adipose tissue index.

### 3.2 Outcomes Within 90 Days After KT

All patients reached a 90‐day analysis (except in case of allograft loss or death < 3 months post‐KT). In the entire cohort, *N* = 72 patients (36%) developed at least one surgical complication up to 90 days after KT **(**Table 2 **)**. *N* = 19 (9.50%) of patients developed intraoperative complications, mainly represented by allograft arterial thrombosis in *N* = 10 (5%) patients and allograft veinous thrombosis in *N* = 4 (2%). Postoperative complications occurred in *N* = 66 (33%) patients and included bleeding/hematoma in *N* = 23 (11.5%), lymphocele in *N* = 13 (6.5%), operative site infection in *N* = 4 (2%), urinoma in *N* = 3 (1.5%) and arterial/veinous thrombosis in *N* = 4 (2%). Death and allograft loss occurred in three (1.5%) and 14 (7%) patients, within 90 days after KT, respectively (Table 2).

**TABLE 2:** Perioperative and postoperative outcomes according to myosteatosis status, within 90 days after KT.

Variables	Whole cohort, *N* = 200	Non myosteatosis patients, *N* = 151	Myosteatosis patients, *N* = 49	*p*
Recipients characteristics				
Male, *N* (%)	123 (62)	89 (59)	34 (69)	0.24
Age, years, mean ± SD	54.8 (±13.8)	53.9 (±13.9)	57.6 (±13.2)	0.11
BMI (kg/m^2^), mean ± SD	25.1 (±4.4)	24.7 (±4.3)	26.4 (±4.5)	**0.024**
Diabetes mellitus, *N* (%)	53 (26.5%)	36 (23.8%)	17 (34.7%)	0.14
Neoplasia history, *N* (%)	28 (14%)	20 (13.3%)	8 (16.3%)	0.64
History of cardiovascular disease, *N* (%)	42 (21%)	27 (17.9%)	15 (30.6%)	0.057
Double KT, *N* (%)	9 (4.5%)	6 (4%)	3 (6.1%)	0.69
KT > 1, *N* (%)	36 (18%)	20 (13.3%)	16 (32.7%)	**0.005**
CT scan data				
SMI (cm^2^/m^2^), mean ± SD	46.9 (±8.9)	47.7 (±8.7)	44.2 (±9)	**0.016**
Muscle density (HU), mean ± SD	30.3 (±8.8)	33.7 (±6.5)	19.7 (±6.1)	**< 0.0001**
VATI (cm^2^/m^2^), mean ± SD	24.7 (±48.5)	19.3 (±13.8)	41.5 (±93.7)	**0.005**
SATI (cm^2^/m^2^), mean ± SD	24.4 (±14.4)	23 (±14.4)	28.9 (±13.6)	**0.012**
Vessel to skin distance (cm), median (IQR)	12.1 (10.3; 14.5)	11.6 (10.1; 13.5)	14.1 (10.8; 15.8)	**0.002**
Outcomes within 90‐days after KT				
Delay graft function, *N* (%)	14 (7%)	9 (6%)	5 (10.2%)	0.34
Surgical complications (CD ≥ 1) up to 90‐days after KT, *N* (%)	72 (36%)	42 (27.8%)	30 (61.2%)	**< 0.0001**
CD ≥ 2 complications, *N* (%)	60 (30%)	32 (21.2%)	28 (57.1%)	**< 0.0001**
CD ≥ 3b complications, *N* (%)	37 (18.5%)	24 (15.9%)	13 (26.5%)	0.096
Intra‐operative complications, *N* (%)	19 (9.5%)	15 (9.9%)	4 (8.2%)	0.71
Allograft arterial thrombosis, *N* (%)	10 (5%)	7 (4.6%)	3 (6.1%)	0.68
Allograft veinous thrombosis, *N* (%)	4 (2%)	4 (2.7%)	0 (0%)	0.25
Vessel dissection, *N* (%)	3 (1.5%)	2 (1.3%)	1 (2%)	0.72
Others, *N* (%)	2 (1%)	2 (1.3%)	0 (0%)	0.42
Post‐operative complications, *N* (%)	66 (33%)	37 (24.5%)	29 (59.2%)	**< 0.0001**
Bleeding/Hematoma, *N* (%)	23 (11.5%)	14 (9.3%)	9 (18.4%)	0.083
Lymphocele, *N* (%)	13 (6.5%)	4 (2.7%)	9 (18.4%)	**0.0001**
Operative site infection, *N* (%)	4 (2%)	1 (0.7%)	3 (6.1%)	**0.018**
Urinoma, *N* (%)	3 (1.5%)	2 (1.3%)	1 (2%)	0.72
Arterial/veinous thrombosis, *N* (%)	4 (2%)	3 (2%)	1 (2%)	0.98
Others, *N* (%)	2 (1%)	2 (1.3%)	0 (0%)	0.82
Death, *N* (%)	3 (1.5%)	2 (1.3%)	1 (2%)	0.57
Allograft loss, *N* (%)	14 (7%)	9 (6%)	5 (10.2%)	0.34

*Note: p*‐value was calculated between myosteatosis and non‐myosteatosis patient groups, using a *χ* ^2^ test for categorial variables and *t*‐test for quantitative variables. *p*‐values < 0.05 are in bold.

Abbreviations: BMI, body mass index; CD, Clavien–Dindo; ECD, extended‐criteria donor; HU, Hounsfield units (density); IQR, interquartile range; KRT, kidney replacement therapy; KT, kidney transplantation; Ly, lymphocyte; MCVE, major cardiovascular event (i.e., coronary heart disease and/or peripheral artery disease and/or history of cerebral stroke); SATI, Subcutaneous adipose tissue index; sCr, serum creatinine concentration; SMI, skeletal muscle mass index; VATI, visceral adipose tissue index.

### 3.3 Risk Factors for Development of CD ≥ 2 Surgical Complication After Kidney Transplantation

Sixty patients (30%) developed at least one CD ≥ 2 complication in the first 3 months after KT (Table 1). In univariate analysis, VSK (cOR: 1.12 for 1 cm increase, 95% CI: 1.01–1.26, *p* = 0.039), MD (cOR: 0.94 for one HU increase, 95% CI: 0.91–0.98, *p* = 0.002) and myosteatosis status (cOR: 4.96, 95% CI: 2.46–9.86, *p* < 0.0001) were significantly associated with the occurrence of CD ≥ 2 postsurgical complication (CD ≥ 2) (Table 3).

**TABLE 3:** Univariable and multivariable logistic regression analysis for CD ≥ 2 surgical complication within 90 days after KT.

Variables	Univariate analysis				Multivariate analysis			
	No/minor complications (CD < 2), *N* = 140	CD ≥ 2 complications, *N* = 60	Crude OR (IC 95%)	*p*	Model 1^b^ Adjusted OR (IC 95%)	*p*	Model 2^c^ Adjusted OR (IC 95%)	*p*
Recipient characteristics								
Age, years, mean ± SD	53.7 (± 14.1)	57.3 (± 12.8)	1.02 (1.00; 1.04)	0.092	1.00 (0.97; 1.03)	0.829	1.02 (0.99; 1.05)	0.21
Diabetes mellitus, *N* (%)	32 (22.9%)	21 (35%)	1.82 (0.94; 3.52)	0.077	1.32 (0.62; 2.80)	0.466	1.31 (0.60; 2.86)	0.71
History of cancer, *N* (%)	23 (16.4%)	5 (8.3%)	0.46 (0.17; 1.28)	0.14	0.37 (0.13; 1.11)	0.076	0.32 (0.10; 1.02)	0.056
History of MCVE, *N* (%)	26 (18.6%)	16 (26.6%)	1.59 (0.78; 3.25)	0.2	1.34 (0.61; 2.92)	0.73	1.14 (0.50; 2.59)	0.75
KT > 1, *N* (%)	22 (15.7%)	14 (23.3%)	1.63 (0.77; 3.46)	0.21	—	—	—	—
CT scan data								
SMI (cm^2^/m^2^), mean ± SD	46.7 (± 8.3)	47.2 (± 10.2)	1.01 (0.97; 1.04)	0.73	—	—	—	—
Muscle density (HU), mean ± SD	31.6 (± 8.3)	27.3 (± 9.1)	0.94 (0.91; 0.98)	**0.002**	0.95 (0.91; 0.99)	**0.028**	—	—
Myosteatosis^a^, *N* (%)	21 (15%)	28 (46.7%)	4.96 (2.49; 9.86)	**< 0.0001**	—	—	4.64 (2.18; 9.90)	**< 0.0001**
VATI (cm^2^/m^2^), mean ± SD	25.5 (± 57.1)	22.9 (± 15.2)	1.00 (0.99; 1.01)	0.73	—	—	—	—
SATI (cm^2^/m^2^), mean ± SD	24 (± 13.8)	25.4 (± 15.7)	1.01 (0.99; 1.03)	0.53	—	—	—	—
Vessel to skin distance (cm), median (IQR)	11.6 (10.2; 13.4)	13.2 (10.5; 15.4)	1.12 (1.01; 1.26)	**0.039**	1.04 (0.91; 1.19)	0.53	1.03 (0.90; 1.18)	0.71

*Note:* In order to avoid multicollinearity effect, ECD criterion variable was not included in the final multivariable because of a strong correlation with recipients age variable.

Abbreviations: CD, Clavien–Dindo; HU, Hounsfield units (density); KT, kidney transplantation; MCVE, major cardiovascular event (i.e., coronary heart disease and/or peripheral artery disease and/or history of cerebral stroke); SATI, subcutaneous adipose tissue index; SMI, skeletal muscle mass index; VATI, visceral adipose tissue index.

^a^ Myosteatosis was defined using specific cut‐off values in KTr from Morel et al. (doi: 10.1002/jcsm.12853).

^b^ Multivariate model including muscle density (UH) as a quantitative variable.

^c^ Multivariate model including muscle density (UH) as a qualitative variable (i.e., myosteatosis status), using specific cut‐off values in KTr from Morel et al. (doi: 10.1002/jcsm.12853).

SATI, VATI and SMI were not significantly associated with the risk of CD ≥ 2 surgical complications in univariate analysis. As these variables are highly sex‐dependent, we tested whether they were associated with the risk of surgical complications in a sex‐adjusted bivariate analysis. No statistical association was found for SATI (*p* = 0.39), VATI (*p* = 0.7) or SMI (*p* = 0.82).

In the first multivariate logistic regression model, including MD but not myosteatosis status, MD was the only independent risk factor for CD ≥ 2 postsurgical complication within 90 days after KT (aOR: 0.95, 95% CI: 0.91–0.99, *p* = 0.028); that is, 5% decreased risk for one HU increase in MD. In the second multivariate model, including myosteatosis status (but not MD), the latter remained the sole independent risk factor for CD ≥ 2 postsurgical complication after KT (aOR: 4.64, 95% CI: 2.18–9.90, *p* < 0.0001) (Table 3). Both multivariate models were adjusted on age, diabetes mellitus status, history of cancer, history of cardiovascular disease and VSK. The area under the curve for the predictive model including only clinical data (age, diabetes mellitus, history of cancer and history of major cardiovascular event) was 0.64 (Figure S1A) and increased to 0.69 (*p* = 0.0073) and 0.72 (*p* ≤ 0.0001) in multivariate models with the addition of MD value or myosteatosis status, respectively (Figure S1B,C).

### 3.4 Outcomes Within 90 Days After KT According to Myosteatosis Status

Forty‐nine patients (24.5%) had CT scan–defined myosteatosis (i.e., low MD) at the time of KT (Table 2). Compared with those with normal MD, myosteatosis patients had higher BMI at KT (26.4 [± 4.5] vs 24.7 [± 4.3} kg/m^2^, *p* = 0.024) and had more often already received at least one kidney transplant (32.7% vs 13.3%, *p* = 0.005). Myosteatosis patients had lower SMI (44.2 ± 9 vs 47.7 ± 8.7 cm^2^/m^2^, *p* = 0.016), higher VATI (41.5 ± 93.7 vs 19.3 ± 13.8 cm^2^, *p* = 0.005) and SATI (28.9 ± 13.6 vs 23 ± 14.4 cm^2^, *p* = 0.012). Within 90 days after KT, patients with myosteatosis experienced more surgical complications (61.2% vs 27.8%, *p* < 0.0001). Intraoperative complications were similar between groups (*p* = 0.71), while postoperative complications were more frequent in myosteatosis patients (59.2% vs 24.5%, *p* < 0.0001), mainly represented by bleeding/hematoma (18.4% vs 9.3%, *p* = 0.083), lymphocele (18.4% vs 2.7%, *p* = 0.0001) and operative site infection (6.1% vs 0.7%, *p* = 0.018) (Table 2).

## 4 Discussion

Our study focuses on the impact of CT scan morphometric data, and more specifically on the impact of myosteatosis on the occurrence of early surgical complications within 90 days of KT. Independently of other morphometric data, only MD values and myosteatosis status were associated with the occurrence of CD ≥ 2 surgical complications.

In our study, the risk of acquiring at least one postoperative complication was 36%, which is slightly lower than in the study by Pinar et al. (42%), which included only on obese and overweight patients (BMI > 25 kg/m^2^) [8]. Tabourin et al. depicted 29.4% of surgical complications in a retrospective study of *N* = 102 KTr, at 1 year after procedure. In this study, low‐MD, but not CSMA, was a risk factor for surgical complications [21].

We used a 3‐month threshold to define early postsurgical complications, as this is a threshold commonly used in surgical morbidity studies, and because complications beyond 3 months are very rarely from surgical origin. In addition, this threshold seemed relevant because it has been demonstrated that there is an excess risk of mortality in the first 3 months of KT [1]. The main CD ≥ 2 surgical complication was postoperative hematoma/bleeding (11.5%), which is similar to the results found in the study by Pinar et al. In the study of Beau et al., hematoma/bleeding prevalence (11%) were also similar to that our study, but postoperative lymphocele were more common (21%) [22].

In univariate analysis, MD and VSK were the only two CT scan data independently associated with CD ≥ 2 surgical complications. VSK has previously been shown to be associated with excess morbidity in obese KTr [8]. Nevertheless, in our study, only MD and myosteatosis status remained independently associated with CD ≥ 2 surgical complications, after adjustment for VSK in two different multivariate models. Pinar et al. did not include the measurement of MD in their study, which involved only overweight KTr, unlike ours.

Moreover, like MD itself, the age‐ and sex‐specific myosteatosis status, in multivariate models using only clinical data, improves the prediction of the occurrence of CD ≥ 2 surgical complications within 90 days of KT, suggesting that myosteatosis could be an interesting tool to better predict surgical complications in KT. It might be important to remind that MD is highly dependent of CT scan instrumentation (including CT scan phase in CT with IV contrast, CT voltage and CT scan slice thickness) [23]. Our age‐ and sex‐specific cutoff for myosteatosis are applicable with the following CT parameters: unenhanced acquisition, 120 kV voltage and 1.25 mm contiguous reconstructed slice thickness [12]. This limitation, and in particular the effect of injection, may disappear in the near future with the development of new methods in clinical practice to quantify myosteatosis with material decomposition on dual‐layer CT scans or dual‐energy CT scans [24].

In our study, myosteatosis patients did not have more intraoperative complications. However, they acquired more postoperative complications, including more lymphocele and surgical site infection. The excess risk of collection/lymphocele in KTr with low‐MD has already been reported by Beau et al. [22]. In this study of 117 patients with a pretransplant CT scan, lymphocele was twice more frequent (55.2% versus 28.4%, *p* = 0.009) in the lowest quartile of MD compared with other patients.

We had previously shown that myosteatosis was associated with long‐term mortality, whereas low muscle mass, measured by CT, was not [12] . These results (i.e., the association between myosteatosis and long‐term mortality, and the absence of a relationship between CT‐measured low muscle mass and mortality) were subsequently confirmed in a large cohort of 992 Chinese kidney transplant recipients [13], and in a cohort of 266 Swedish KTr [14]. It therefore seems that abnormal muscle quality may be a more important morbidity and mortality factor than low muscle mass assessed by CT in KT. It should also be noted that the method used to assess muscle mass may influence study results. For example, Gaillard et al. [25] showed that urinary creatinine excretion, a surrogate biomarker of total skeletal muscle mass, was associated with KTr mortality.

Studies evaluating the clinical consequences of excess visceral and subcutaneous fat measured by CT in KTr remain scarce. To our knowledge, visceral fat measured by CT scan or dual‐energy X‐ray absorptiometry scan has only been shown to predict the occurrence of posttransplant diabetes mellitus [26, 27]. Pinar et al. also did not find any association between postsurgical complications, and visceral fat or subcutaneous fat measured by CT in obese and overweight patients [8].

The causes of myosteatosis are multifactorial. The determinants of myosteatosis in the general population include age [28], insulin resistance [29], physical inactivity [30] and inflammation [31]. Mitochondrial dysfunction, leading to reduced mitochondrial lipid oxidation, could be key to muscle fat accumulation [32]. Advanced age, insulin resistance, sedentary lifestyle and inflammation are common conditions in CKD patients. It is also possible that the accumulation of uremic toxins promotes mitochondrial dysfunction in CKD patients [33], leading to a CKD‐specific mechanism of myosteatosis. Chronic acidosis could also be an indirect factor leading to myosteatosis in CKD patients. Indeed, metabolic acidosis, which is a common complication of severe CKD, is a major factor promoting insulin resistance [34], which in turn may lead to fat accumulation in the muscles.

The pathophysiological link between myosteatosis and the occurrence of postoperative complications remains to be clarified. Myosteatosis, which is associated with reduced physical capacity, particularly in patients with chronic kidney disease [35], could be at least partially overlapped with the frailty syndrome, defined as the presence of 3 or more of the following characteristics: unintentional weight loss, weakness, slowness, exhaustion and low physical activity [36]. The diagnosis of myosteatosis could therefore lead to identifying patients in a state of increased vulnerability due to reduced reserves, impairing their ability to adapt to acute stress factors such as surgery. It should also be noted that several of the determinants of myosteatosis have been shown to be associated with the risk of surgical complications. For example, insulin resistance is associated with an increased risk of major postsurgical complications, irrespective of the patient's diabetic status [37]. The same applies to physical inactivity [38] and advanced age [39]. These individual risks could be amplified in a deleterious synergy, in the context of myosteatosis.

While we depicted, in a previous article on this same cohort, that myosteatosis was a risk factor for long‐term posttransplant mortality [12], we did not find significant difference in mortality according to myosteatosis status within 90 days posttransplant. This could be explained by the low number of events (*n* = 3). In the same way, the higher rate of allograft loss in patients with myosteatosis (10% vs 5%) did not reach statistical significance, maybe because of the low number of events in the whole cohort (*n* = 14).

Our study has several weaknesses in addition to its retrospective, monocentric design. The overall size of the population is limited. Another limitation is the time span between CT scan acquisition and surgery (up to 1 year), which means that for some patients, morphometric data may have changed between the time of the CT scan and that of the KT. Our study focused on surgical complications classified as Grade 2 or higher according to the Clavien–Dindo classification. While these Grade 2 complications do not require interventional therapy, they constitute the bulk of surgical morbidity in kidney transplantation, where surgical or radiological reinterventions are relatively rare. In our study, less than one in five patients had a CD ≥ 3b complication (no complications classified as CD3a). These CD ≥ 3b complications were almost twice as frequent in patients with myosteatosis compared with those without myosteatosis, but the difference was not statistically significant, possibly due to the small number of events. We also did not collect the proportion of patients on immediate intraoperative or postoperative anticoagulant therapy in our study, which might limit the interpretation of the statistical relation between hematoma, lymphocele and myosteatosis status. Several surgical operators performed the kidney transplantations during the study period. They all came from the same centre and used the same technique, but it is possible that there were differences in experience between operators and that the times of surgery, sometimes at night, may have had an impact on the complication risk. Unfortunately, we were unable to retrieve these data and include them in our multivariate models. Another important piece of data that we were unable to collect in this retrospective study is the level of physical activity of patients, which also constitutes a weakness of the study since physical inactivity may be one of the determinants of myosteatosis, which in turn alters physical performance in CKD patients [35].

In conclusion, low‐MD, a surrogate of myosteatosis, measured by CT scan is independently associated with the occurrence of a postoperative complication within 90 days of KT surgery and might be a tool of interest in order to better predict surgical complications in KTr. Identification of such risk factor should lead to more careful monitoring after kidney transplant surgery and may require specific pre‐transplant information for future KTr. If the association between surgical complication risk and myosteatosis is confirmed in other large‐scale studies, the question of therapeutic interventions to reverse myosteatosis and/or the clinical consequences of myosteatosis, such as pre‐habilitation programs for patients with myosteatosis awaiting KT, will arise.

## Conflicts of Interest

The authors declare no conflicts of interest.

## Supporting information


**Figure S1A** Area under the ROC curve (AUROC) of the Cox multivariable model, using only clinical data (age, diabetes mellitus, history of cancer and history of major cardiovascular event).
Figure S1B: Area under the ROC curve (AUROC) of the Cox multivariable model, using muscle density as a continuous variable.
Figure S1C: Area under the ROC curve (AUROC) of the Cox multivariable model, using muscle density as a categorical variable (i.e., myosteatosis status).
