# Supplementary material for: Myosteatosis as a New Risk Factor of Surgical Complications in Kidney Transplant Recipients: A Retrospective Study
Source: J Cachexia Sarcopenia Muscle. 2025 Apr 30;16(3):e13746. doi: 10.1002/jcsm.13746 (PMC12041939; doi:10.1002/jcsm.13746)
Supplement: Supplementary file 1 — Figure S1A Area under the ROC curve (AUROC) of the Cox multivariable model, using only clinical data (age, diabetes mellitus, history of cancer and history of major cardiovascular event). Figure S1B: Area under the ROC curve (AUROC) of the Cox multivariable model, using muscle density as a continuous variable. Figure S1C: Area under the ROC curve (AUROC) of the Cox multivariable model, using muscle density as a categorical variable (i.e., myosteatosis status). [file JCSM-16-e13746-s001.docx]

**Digital supplemental content**

**Supplemental Figure 1A: Area Under the ROC curve (AUROC) of the Cox multivariable model, using only clinical data (age, diabetes mellitus, history of cancer and history of major cardiovascular event).**

**
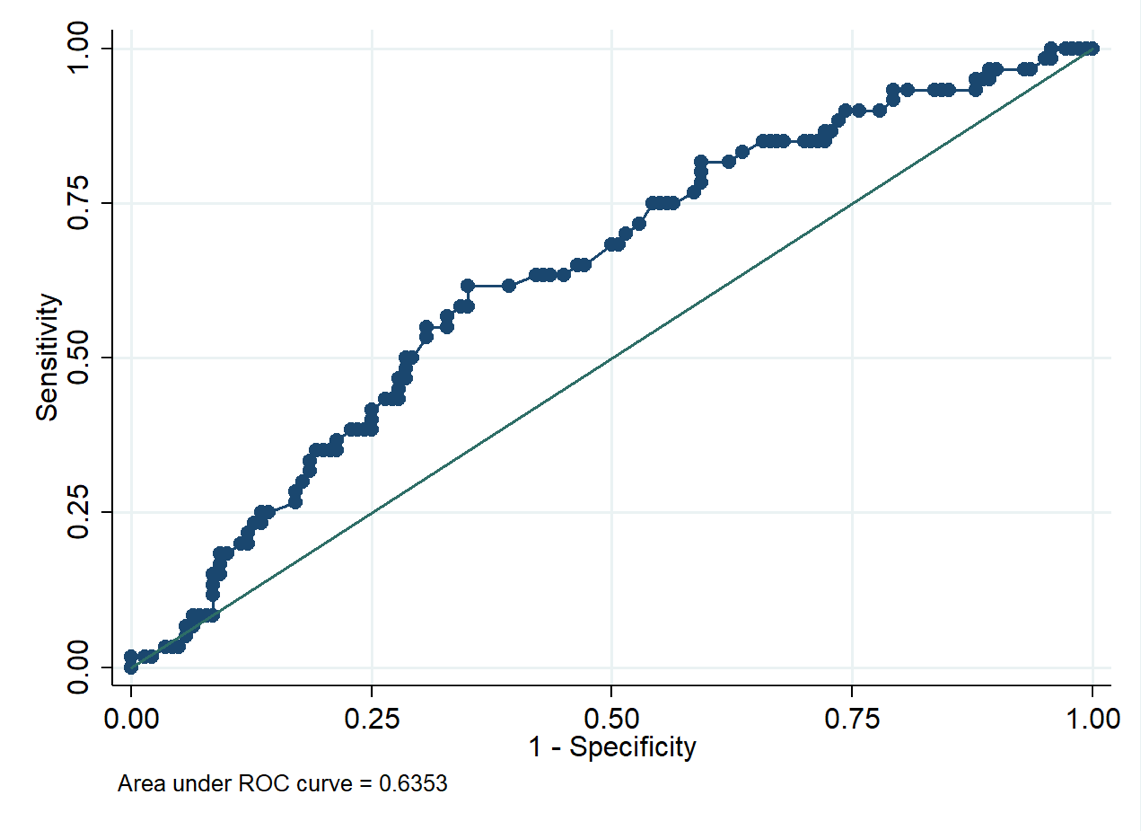
**

**Supplemental Figure 1B: Area Under the ROC curve (AUROC) of the Cox multivariable model, using muscle density as a continuous variable**

**Supplemental Figure 1C: Area Under the ROC curve (AUROC) of the Cox multivariable model, using muscle density as a categorical variable (*i.e* myosteatosis status)**
